# In this issue: Volume 114, Issue 10, October 2023

**DOI:** 10.1111/cas.15969

**Published:** 2023-09-20

## Targeting the loss of cGAS/STING signaling in cancer

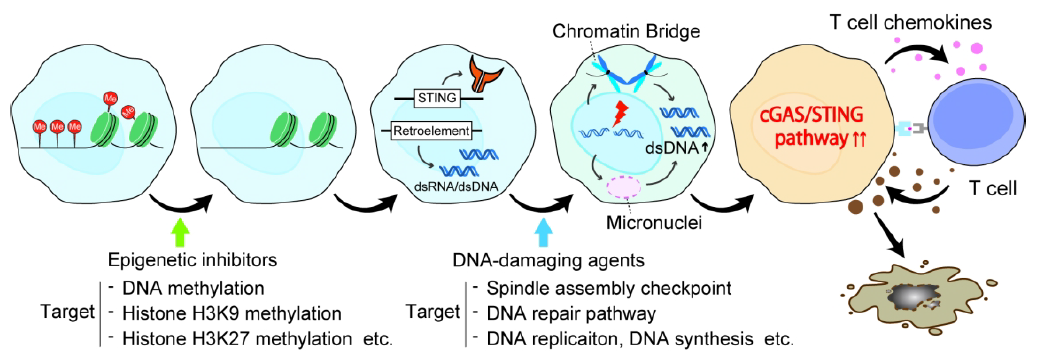

Our bodies have an innate immune system that acts as the first line of defense against pathogenic bacteria and viruses. Stimulator of interferon genes or STING is a protein that plays an important role in this innate immune system. STING is activated by messengers which are produced by the cGAS enzyme on sensing abnormal levels of cytoplasmic double stranded DNA (dsDNA) on microbial or viral invasion. Activated STING triggers a cascade of events, leading to the production of type I interferons (IFNs) and other immune molecules, that play a role in both innate and adaptive immunity by killing infected cells. Cancer cells often have high levels of aberrant cytoplasmic DNA, which can also trigger an immune response leading to cell death. However, cancer cells evade this fate by suppressing the cGAS/STING pathway.

In this review, researchers discussed different strategies that can help in regenerating anti‐tumor immunity through reactivation of the cGAS/STING pathway.

They identified that synthetic STING agonists are promising candidates for cGAS/STING reactivation. These molecules often resemble cyclic dinucleotides (CDNs) and are effective in inducing anti‐tumor immunity in animal models. However, poor cell membrane permeability blocks the effective delivery of synthetic STING agonists to the cytoplasm in cancer cells. Another challenge is the limited accessibility of intratumoral injection, which makes it difficult to deliver the drug to the tumoral cells. Non‐nucleotide‐based STING agonists are being developed for systemic administration, but they carry safety risks due to the potential for causing adverse effects.

Another strategy is to use DNA‐damaging agents, such as chemotherapy drugs and targeted therapies that inhibit DNA repair pathways. These agents can cause the accumulation of cytoplasmic DNA, activating cGAS to produce the endogenous STING agonist. Cancer cells often have epigenetically silenced cGAS and STING genes, which means that these genes are turned off. Epigenetic inhibitors are drugs that can reverse this silencing, making the cancer cells more responsive to STING agonists. Therefore, combining epigenetic inhibitors with STING agonists can be a more effective way to activate the cGAS/STING pathway and kill cancer cells.

In conclusion, to effectively utilize the cGAS/STING signaling pathway for cancer immunotherapy, it is important to understand the mechanisms by which cGAS/STING signaling is suppressed in cancer cells. With further research, the strategies highlighted in this review can open novel therapeutic avenues for cancer immunotherapy.

https://onlinelibrary.wiley.com/doi/10.1111/cas.15913

## Personalized medicine with germline pathogenic variants: Importance of population‐ and region‐wide evidence

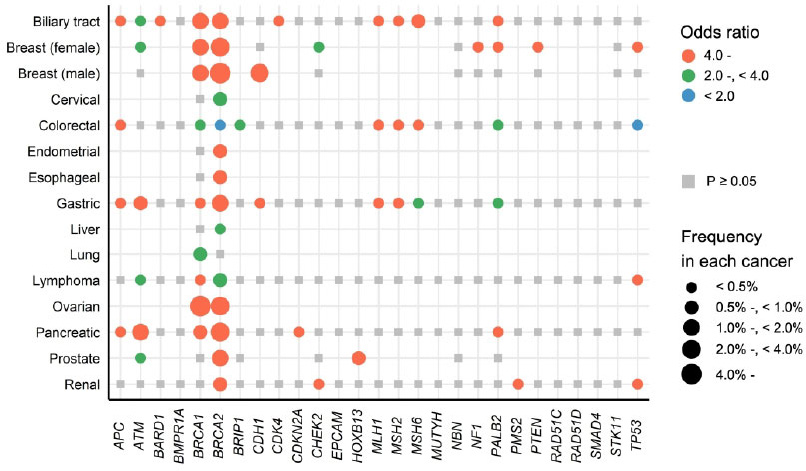

Germline pathogenic variants (GPVs) are genetic mutations in germ cells that can be inherited by successive generations. Such GPVs in cancer‐predisposing genes may lead to an increase in cancer susceptibility for individuals. Over the past three decades, evidence of GPV‐linked cancer risk has led to many developments in the field of personalized cancer medicine. Moreover, developing treatment, risk reduction, and surveillance strategies based on germline variants could improve cancer management for at‐risk and affected individuals. However, existing evidence in this regard mainly originates from studies on European populations. This makes it difficult to apply these strategies for Japanese population, who may report differences in the prevalence of cancer‐related GPVs.

To address this, researchers previously analyzed data from more than 100,000 individuals from Japan to explore cancer risk associations with germline variants in cancer‐predisposing genes. They newly found high prevalence of GPVs in BRCA1 and/or BRCA2, particularly in biliary tract, esophageal, and gastric cancers. On the contrary, the prevalence of GPVs in other cancer‐associated genes like CDH1 and STK11 was low in Japanese female breast cancer, which are known to be associated with risk of female breast cancer in the National Comprehensive Cancer Network guidelines. In this review, the researchers summarized the recent advances in personalized medicine and discussed the potential use of GPVs for cancer management.

They report that the identification of association between disease onset and GPVs has led to novel treatment strategies. For instance, the discovery of BRCA1/2's role in homologous recombination repair prompted the development of PARP inhibitors that have been particularly effective at combating breast, ovarian, pancreatic, and prostate cancers.

Preemptive surgical measures, such as bilateral mastectomy for breast cancer, salpingo‐oophorectomy for ovarian cancer, colectomy for colorectal cancer, and gastrectomy for gastric cancer are now discussed for reducing the cancer risk in GPV carriers. However, evidence for levels of recommendation, associated side effects, impact of these measures on quality of life, and ethical questions, should be factored in. Chemoprevention is yet another way to reduce GPV‐related cancer risk. The use of aspirin for colorectal cancer can also lower cancer risk in individuals with Lynch syndrome. Risk reduction may be achieved by managing external factors like Helicobacter pylori infection, which are associated with an excess risk of gastric cancer among GPV carriers in homologous recombination genes.

The review thus summarizes recent advances that can be helpful for personalized medicine and highlights the potential for expanding the evaluation of germline variants in diverse populations and regions for cancer management.

https://onlinelibrary.wiley.com/doi/10.1111/cas.15922

## Single‐cell RNA‐seq reveals a microenvironment and an exhaustion state of T/NK cells in acute myeloid leukemia

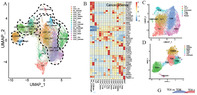

Acute myeloid leukemia (AML) is a complex blood cancer marked by abnormal cell growth in the bone marrow. Despite progress in AML treatment over the years, disease relapse remains a challenge, underscoring the need for new therapeutic approaches.

In this context, immune‐based therapies have shown promise against other types of blood cancer. However, their effectiveness in treating AML has been limited, partly due to the hostile conditions within the AML tumor microenvironment (TME). Within the TME, crucial immune system cells, such as T cells and natural killer (NK) cells can become exhausted, losing their ability to kill cancer cells. While the exact mechanisms behind this exhaustion have remained unclear, a deeper understanding of the association between TME and AML is certainly needed.

In this study, researchers used single‐cell RNA sequencing (scRNA‐seq) technology to analyze the composition of TME in AML. They constructed a comprehensive map of the TME containing data from 128,688 cells from AML patients and healthy donors. In this TME landscape, nine major cell types were identified. Further, the researchers created a detailed map of the TME, delineating these nine distinct major cell types.

Notably, malignant AML cells were found to co‐cluster with hematopoietic stem cells (HSC)/progenitor cells in the TME. HSCs give rise to all blood cell types, and progenitor cells are partially differentiated into blood cell precursors. Finding AML cells in the same cluster as HSC/progenitor cells suggests that these cells may be involved in the development of AML.

Further, the TME was found to be infiltrated by a variety of cells, including myeloid‐derived suppressor cells (MDSCs), regulatory T cells (Tregs), and tumor‐infiltrating lymphocytes (TILs). These cells can produce immunosuppressive factors, which can lead to the exhaustion of T and NK cells. Exhausted T cells might develop from tissue‐resident memory T cells (TRM) within the AML TME.

Further, they observed higher expression of checkpoint molecules like TIGIT, KLRC1, and CD96 in exhausted T and NK cells, which hindered the ability of these cells to recognize and eliminate cancer cells. The expression of certain genes, such as TOX and TCF‐1, was linked to T and NK cell exhaustion. Addressing their expression may have the potential to reverse or prevent exhaustion. Moreover, despite exhaustion, NK cells in AML patients can potentially kill cancer cells via an “activation‐dependent exhaustion expression program,” offering new treatment possibilities.

In summary, understanding the AML TME and its impact on immune cell dynamics could provide valuable insights for improving AML immunotherapies in the future.

https://onlinelibrary.wiley.com/doi/10.1111/cas.15932

